# Recent advances in photothermal therapy-based multifunctional nanoplatforms for breast cancer

**DOI:** 10.3389/fchem.2022.1024177

**Published:** 2022-09-19

**Authors:** Jingjun Sun, Haiyan Zhao, Weixuan Xu, Guo-Qin Jiang

**Affiliations:** ^1^ Department of Surgery, the Second Affiliated Hospital of Soochow University, Suzhou, China; ^2^ Department of Breast Surgery, Affiliated Maternity and Child Health Care Hospital of Nantong University, Nantong, China; ^3^ Department of Breast Surgery, Shanghai Changning Maternity and Infant Health Hospital, East China Normal University, Shanghai, China

**Keywords:** breast cancer, photothermal therapy, accurate therapy, targeted delivery platform, NIR dye, combination therapeutic strategy

## Abstract

Breast cancer (BC) is one of the most common cancers in women worldwide; however, the successful treatment of BC, especially triple-negative breast cancer (TNBC), remains a significant clinical challenge. Recently, photothermal therapy (PTT), which involves the generation of heat under irradiation to achieve photothermal ablation of BC with minimal invasiveness and outstanding spatial–temporal selectivity, has been demonstrated as a novel therapy that can overcome the drawbacks of chemotherapy or surgery. Significantly, when combining PTT with chemotherapy and/or photodynamic therapy, an enhanced synergistic therapeutic effect can be achieved in both primary and metastatic BC tumors. Thus, this review discusses the recent developments in nanotechnology-based photothermal therapy for the treatment of BC and its metastasis to provide potential strategies for future BC treatment.

## Introduction

Breast cancer (BC) is one of the most common malignancies in women worldwide, and the 5-year survival rate of patients with stage III BC is approximately 50%, with an average of only 4.9 years (Gradishar et al., 2020; Siegel et al., 2021; Afzal et al., 2022; An et al., 2022; Luo et al., 2022). Due to genetic susceptibility, environmental factors, lifestyle, etc., there are significant differences in the incidence and mortality of BC across countries (Ginsburg et al., 2017; Hou et al., 2021). Previous studies have demonstrated that about 0.16 million patients were affected by BC in the United States in 2017 (Torre et al., 2016; Li et al., 2020a). Since 2020, there have been 2.3 million new cases of BC worldwide every year, and the total number of BC patients is predicted to increase by 50% by 2040 (Dias et al., 2021; Shao and Varamini, 2022).

At the molecular level, BC is mainly classified into four different subtypes, luminal A, luminal B, HER2-enriched, and triple-negative breast cancer (TNBC), based on the expression of epidermal growth factor receptor 2 (HER2), estrogen receptor alpha (ER), and progesterone receptor (PR) (Dai et al., 2015; Prat et al., 2015; Azamjah et al., 2019). Clinically, ER- and/or PR-positive and HER2-negative tumors are defined as luminal A, ER- and/or PR-positive, and HER-positive with high Ki67 expression is luminal B; HER2 overexpression with ER- and PR-negative is HER2-enriched; and triple-negative breast cancer (TNBC) is a subtype lacking ER, PR, and HER2 expression but with high Ki67 expression (Cancer Genome Atlas, 2012; Nagarajan and McArdle, 2018; Roswall et al., 2018).

Patients diagnosed with different BC subtypes exhibit high heterogeneity in prognosis. In addition to apocrine carcinoma, lobular carcinoma, and metaplastic carcinoma, approximately 90% of TNBC cases exhibit ductal carcinoma infiltration (Gonzalez-Angulo et al., 2011; Mendes et al., 2015; Salgado et al., 2015). Although the 5-year survival rate of patients with TNBC is more than 60%, the median survival of patients with advanced TNBC is only 1 year (Pal et al., 2014; Pawar and Prabhu, 2019). Aside from the lack of targeted therapy, patients with TNBC usually have a higher risk of metastasis with poorly differentiated grades among all BC subtypes (Chen et al., 2022a; Chen et al., 2022b; Tan et al., 2022). Even with the development of magnetic resonance imaging and positron emission tomography with screening programs, the precise diagnosis of TNBC at early stages remains a significant challenge in the clinic because of the aggressiveness and rapid pathological process into advanced stages. These factors significantly reduce the survival rates of patients with TNBC. Therefore, it is important to find an optimal strategy with desirable therapeutic effects for treating TNBC and improving patient prognosis.

## Photothermal therapy

**FIGURE 1:**
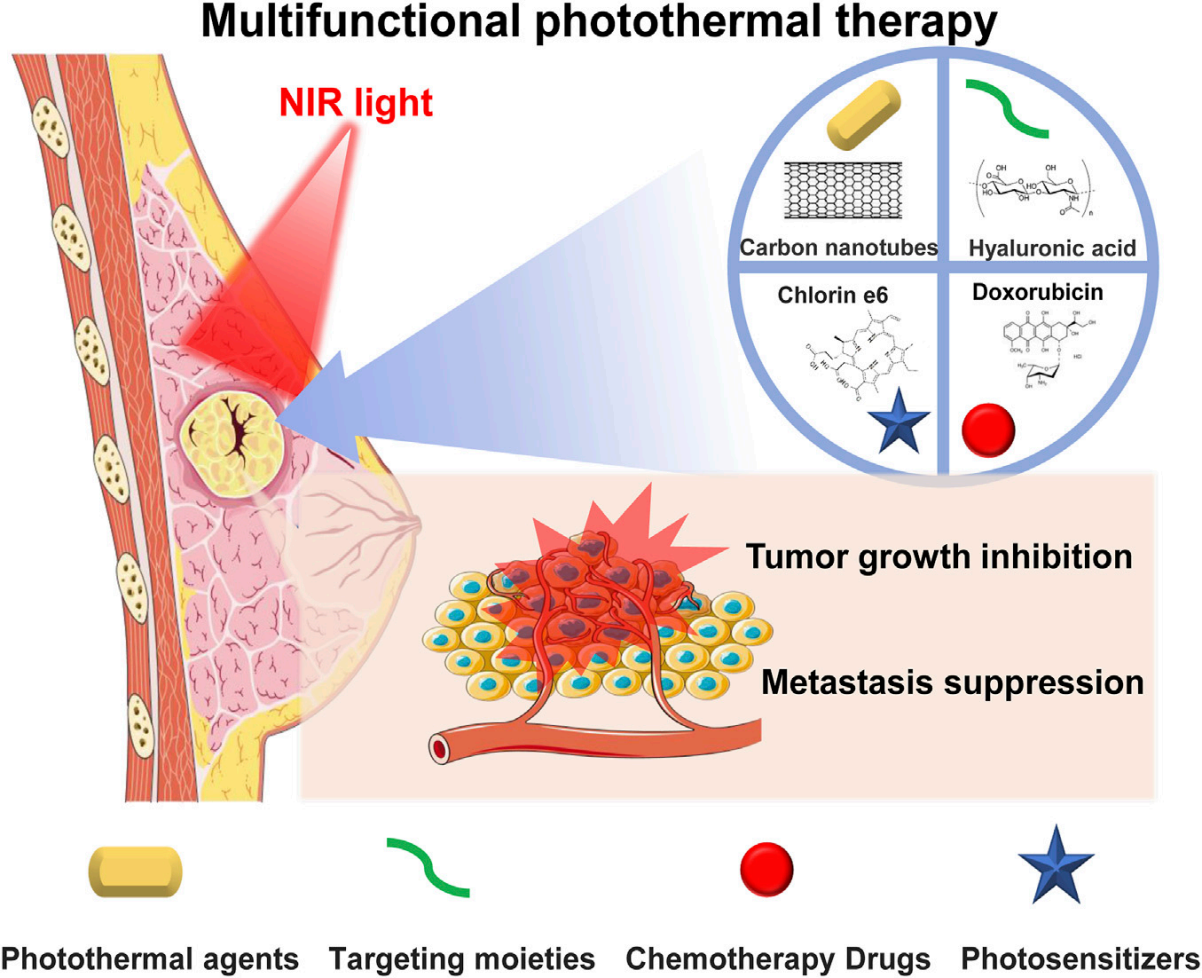
Photothermal therapy combined with chemotherapy or/and photodynamic therapy to achieve a synergistic therapeutic effect to inhibit breast cancer primary tumor and metastasis.

The current therapeutic approaches for BC mainly consist of chemotherapy, hormone therapy, and surgery (Aghanejad et al., 2013; Kadkhoda et al., 2022; Zhong et al., 2022). However, disadvantages such as adverse side effects for patients, drug resistance, and residual tumor cells greatly limit their therapeutic effect and may lead to cancer recurrence (Waks and Winer, 2019; Kadkhoda et al., 2021). In addition, surgery can only remove solid tumors from patients in the early stages, and surgical trauma can induce systemic inflammatory responses to promote micrometastatic growth. Photothermal therapy (PTT) has been demonstrated to be an emerging therapeutic method with low toxicity, minimal invasiveness, and outstanding spatial–temporal selectivity, which could overcome these drawbacks (Shakil et al., 2019; Mao and Liu, 2020; Peng et al., 2021). By irradiating photothermal agents under near-infrared (NIR) light, hyperthermia can be triggered to kill cancer cells in target tissues by energy transfer through electron–phonon and electron–electron relaxation of photothermal agents that increase temperature, with both primary tumors and early local metastasis being potential targets. PTT can effectively suppress BC by activating apoptosis, autophagy, or suppressing cell signaling to induce cell death with a shorter treatment time, which reduces patient pain and possesses desirable therapeutic effects with fewer side effects (Kadkhoda et al., 2022). Moreover, when combining PTT with chemotherapy and/or photodynamic therapy, an enhanced synergistic therapeutic effect can be achieved in both primary and metastatic BC tumors (Zhou et al., 2015b; Guo et al., 2015; Lin et al., 2015; Liu et al., 2019; Deng et al., 2021) (Figure 1).

## Photothermal therapy-triggered apoptosis

Apoptosis is a highly regulated process of cell death distinct from necrosis, which involves intracellular signals, such as DNA damage or growth factor deprivation, and extracellular signals produced during the immune response to cell damage or infection (Pfeffer and Singh, 2018). It has been reported that PTT can also suppress tumors by initiating cell apoptosis *via* the apoptosis internal pathway, which makes PTT one of the most reliable and powerful methods for cancer treatment (Li et al., 2022).

A recent study has shown that gold nanoparticles (AuNPs) with epidermal growth factor receptor-targeting antibodies may serve as a promising tool for NIR photothermal therapies for cancer *via* apoptotic pathways. EGFRmAb-conjugated AuNPs exhibited high selectivity and cytotoxicity against cancer cells, where they entered the nucleus. NIR irradiation of AuNPs induced cell apoptosis and DNA damage by inhibiting the PI3K/AKT/mTOR pathway and upregulating the double-strand DNA break repair proteins (Zhang et al., 2018).

Regarding breast cancer treatment, Shang et al. previously reported an AuNP-based theranostic agent synthesized by sequentially coating colloidal polystyrene spheres with polydopamine and AuNPs. This colloidal polydopamine (PDA)/Au agent can not only induce photothermal ablation of breast cancer cells under NIR light but also provide significant enhancement for ultrasound imaging in oncology (Shang et al., 2020).

Shang et al. reported that a combination therapy of PTT and chemotherapy is a promising strategy for breast cancer treatment by equipping gold nanorods (GNRs) with hyaluronic acid/chitosan and doxorubicin *via* a Schiff base linkage. The hyaluronic acid corona improved the stability of the polysaccharide-based nanoplatforms and allowed for the effective targeting of the CD44 receptor in MCF-7 breast cancer cells. Doxorubicin is released in acidic microenvironments in the tumor to promote pH-responsive drug release behavior. The combined chemo-photothermal therapy exhibited better therapeutic effects than either PTT or doxorubicin individually (Xu et al., 2019).

The overexpression of epidermal growth factor receptor (EGFR) in TNBC enables novel EGFR-targeted therapies for TNBC. Based on clinical ultrasound and photoacoustic imaging, Zhang et al. conjugated gold nanorods with an anti-EGFR antibody to allow for the accurate detection of solid primary tumors and lymph node metastases of TNBC *in vivo* with efficient NIR photothermal therapy. PTT using anti-EGFR-conjugated gold nanorods can also activate apoptosis in TNBC by upregulating HSP70 and cleaved caspase-3 while suppressing Ki-67 and EGFR (Zhang et al., 2017).

Although PTT can induce cell death pathways, such as apoptosis and necrosis, apoptosis has been regarded as a more effective method to remove cancer cells than necrosis, as it protects the plasma membrane integrity of cells and avoids inflammation. Xing et al. developed coral-shaped Au nanostructures (Au NCs) with a high surface-to-volume ratio to provide better photothermal conversion in the PPT treatment of breast cancer. Under low-power NIR irradiation for 15 min, Au NCs induced apoptosis in MCF-7 cells by upregulating Bax nuclear-encoded proteins and suppressing Bcl-2 protein expression in the apoptotic pathway, which successfully inhibited cancer recurrence *in vivo* (Xing et al., 2019).

These findings demonstrate that functionalizing photothermal agents with targeted moieties and combining PTT with other effective therapies are gaining increasing attention and have great potential for treating BC.

## Photothermal therapy on BC metastasis

Metastasis, including bone, lung, liver, and brain metastases, in BC patients generally results in a poor prognosis, besides removal of the primary tumor (Gong et al., 2017; Ling et al., 2021; Zuo et al., 2022). Among all BC cases, 50% of BC in patients progressed to liver metastases, and there is still no effective therapy to treat liver metastatic estrogen receptor α (ERα)-positive breast cancer, with these patients showing correspondingly poor outcomes (Boudreau et al., 2021; Rashid et al., 2021); however, despite considerable effort, the biological mechanism of metastasis in BC remains unclear. Studies have shown that patients with the HER2-positive or triple-negative subtypes have a significantly higher risk of developing liver metastases than patients with the HR+/HER2-subtype (Kennecke et al., 2010; van de Water et al., 2012; Soni et al., 2015). The reason for this phenomenon may be that HER2 can activate the chemokine receptor CXCR4 and increase the expression of fibroblast growth factor homologous factor (FGF13) to promote the progression of liver metastasis through the CXCL12/CXCR4 pathway in TNBC (Zlotnik et al., 2011; Johnstone et al., 2020). In contrast, long noncoding RNAs, a group of RNAs over 200 nucleotides in length but which lack the ability to code for proteins, have been recently demonstrated to promote invasion and metastasis of cancer cells by initiating the epithelial to the mesenchymal transition process in BC progression (Zhou et al., 2016; Hussen et al., 2021; Hussen et al., 2022). Moreover, as cell adhesion molecules (CAMs) play an important role in cancer cell invasion and metastasis, one study showed that CD44 could regulate PCF11 via the MAPK/ERK pathway or TGF-β signaling pathway to promote metastasis in BC (Ouhtit et al., 2013; Al-Mansoob et al., 2022).

Significantly, hyperthermia has been reported to have a strong capacity to suppress the expression of metastasis-related factors, such as vascular epithelial growth factor, metalloproteinase, and TGF-β1, and hinder the invasion and metastasis of cancer cells (Okuno et al., 2013; Zhou et al., 2015a; Zou et al., 2016; Alamdari et al., 2022). He et al. developed a photothermal nanoplatform by assembling 1,1-dioctadecyl-3,3,3,3-tetramethylindotricarbocyanine iodide (DIR) into an amphiphilic polymer of poly(ethylene glycol)-block-poly (2-diisopropylmethacrylate) for PTT on BC metastasis. This photothermal nanoplatform exhibited strong light-absorbing capability upon 808 nm NIR irradiation, producing hyperthermia to suppress the invasion of metastatic BC cells. Compared to DIR alone, the nanoplatform showed significantly improved accumulation, which facilitated the PTT in inhibiting tumor progression and metastasis *in vivo* (He et al., 2015).

By simple mixing of an FDA-approved NIR dye, indocyanine green (ICG), with human serum albumin (HSA) and paclitaxel (PTX), biocompatible photothermal nanoparticles can be self-assembled. The ICG moiety can generate mild photothermal heating to improve intracellular uptake in 4T1 murine breast cancer cells upon NIR irradiation to enhance the synergistic therapeutic efficacy. This HSA-ICG–PTX photothermal nanoparticle has excellent capacity to treat primary tumors and, more importantly, suppress lung metastasis (Chen et al., 2015).

### Synergistic therapy of photothermal therapy/chemotherapy

Therapies combining PTT with other treatments, such as photodynamic therapy, chemotherapy, and exosome therapy, have attracted the interest of many researchers worldwide owing to their potential to further improve the PTT-based treatment for cueing BC and distant metastasis (Zhou et al., 2015b; Guo et al., 2015; Lin et al., 2015; Liu et al., 2019; Deng et al., 2021). For example, Tian et al. developed a nanoparticle system by loading ICG dye and DOX into porous silicon nanoparticles to achieve chemo-photothermal therapy to inhibit the growth and metastasis of BC. This drug delivery system was equipped with a tumor cell-derived exosome membrane to enhance its accumulation in tumor sites and intracellular uptake by cancer cells. The heat produced by biomimetic nanoparticles under NIR irradiation can effectively accelerate the release of DOX and facilitate tumor ablation to suppress tumor growth and metastasis in a BC tumor-bearing mouse model. This nanosystem may serve as a promising tool for combination therapies for BC (Tian et al., 2020).

Ma et al. developed another drug delivery system based on ICG, HSA, and DOX. This nanoplatform can generate mild hyperthermia to enhance the cellular uptake in cancer cells. Furthermore, it can successfully induce T-cell responses by increasing T-cell permeability and activating cytotoxic T cells to suppress distant metastasis of BC, which demonstrates a promising T-cell response-enhanced chemo-photothermal therapy against BC (Ma et al., 2020).

As P-selectin proteins overexpressed on platelet membranes can bind to CD44 receptors on BC cells, Ye et al. coated PLGA-based photothermal nanoparticles with platelet membranes to enhance the impact of both PTT and chemotherapy. ICG serves as a PTA to provide NIR-induced hyperthermia, while DOX is a chemotherapeutic agent. Favored by the interaction between CD44 receptors on BC cells and P-selectin on nanoplatelets, these PLGA nanoparticles could easily accumulate in MDA-MB231 breast cancer cells and exhibited a strong capacity to inhibit breast cancer metastasis *in vivo* (Ye et al., 2019).

### Synergistic therapy of photothermal therapy/photodynamic therapy

Photodynamic therapy (PDT) has been demonstrated as a minimally invasive method to treat various types of cancer, including breast cancer, melanoma, and lung cancer (Tang et al., 2021; Ding et al., 2022; Jia et al., 2022; Obaid et al., 2022; Pan et al., 2022; Zhang et al., 2022). The photosensitizers can be activated under a light source to generate reactive oxygen species (ROS) to kill cancer cells. In particular, PDT can avoid systemic toxicity with fewer side effects than chemotherapy due to its high spatiotemporal selectivity (Li et al., 2018; Zhang et al., 2019; Nguyen et al., 2022). To achieve synergistic therapeutic effects with different mechanisms for eliminating breast cancer, combination therapies involving both PDT and PTT have been developed (Su et al., 2015; Lee et al., 2022; Xu et al., 2022).

For instance, Li et al. developed a Lyp-1 (CGNKRTRGC)-modified micellar system by stabilizing negatively charged NIR dye-IR820 with cationic PCL-grafted poly (ethylene imine) to produce a combination PTT/PDT therapy for breast cancer. Lyp-1 peptides precisely target the p32 protein overexpressed on breast cancer cells to enhance the targeting effect of the micellar system. Importantly, PTT/PDT/chemotherapy exhibited excellent inhibition of growth and metastasis in a 4T1 cancer model in BALB/c nude mice (Li et al., 2016).

To overcome the drawbacks of drug resistance and insufficient targeting ability in chemotherapy for TNBC, Li et al. developed chlorin e6 (Ce6)-functionalized AuNPs for synergistic PTT/PDT for the treatment of TNBC. By further equipping nanoparticles with cRGD peptides and triphenylphosphonium cationic moieties, the nanosystem can specifically target TNBC cells and mitochondria to enhance their accumulation in tumors, which facilitates hyperthermia and ROS generation under NIR irradiation for synergistic anti-TNBC effects in mice (Li et al., 2021).

Lung metastasis is one of the main causes of breast cancer treatment failure (Guo et al., 2022; Houthuijzen and de Visser, 2022). To achieve desirable metastasis inhibition, Li et al. synthesized theranostic gold nanostars with polydopamine (PDA) and Ce6 conjugation for precise PTT and PDT to inhibit 4T1 tumors and their lung metastasis. These theranostic gold nanostars exhibited excellent stability and photothermal conversion and further possessed simultaneous photoacoustic imaging for an accurate therapeutic strategy (Li et al., 2020b).

## Conclusion and future perspectives

Many achievements have been made in the development of intelligent nanosystems with PTT, even when combined with other effective therapies to enhance the therapeutic effect against breast cancer. However, several challenges limiting its efficacy still remain, such as NIR penetration depth, the toxicity of photothermal agents, and thermal resistance. To develop photothermal agents, the cross-sectional area of absorption and photothermal conversion efficiency should be increased. Long-term biosafety, stability, and targeting ability should also be considered to improve the efficacy *in vivo*. Thus, combination therapy may enable the application of photothermal agents at lower doses; however, the interaction and influence between different components have not yet been elucidated. Furthermore, current strategies have mainly focused on directly killing breast cancer cells in both primary tumors and metastases, whereas the tumor microenvironment plays a significant role in tumor progression. Successful regulation of immune responses in the TME may greatly improve outcomes. In addition, underlying mechanisms and pathways involved in PTT-triggered apoptosis or antimetastasis should be more clearly evaluated. In summary, the design of multifunctional PTT nanotools has been shown to be a promising direction for the successful treatment of BC in the future.
